# Socioeconomic position and participation in colorectal cancer screening

**DOI:** 10.1038/sj.bjc.6605962

**Published:** 2010-10-19

**Authors:** B L Frederiksen, T Jørgensen, K Brasso, I Holten, M Osler

**Affiliations:** 1 Research Centre for Prevention and Health, Capital Region of Denmark, Glostrup University Hospital, Building 84/85, DK-2600 Glostrup, Denmark; 2 Institute of Public Health, Department of Social Medicine, University of Copenhagen, 1014 Copenhagen K, Denmark; 3 Department of Urology, University Hospital of Copenhagen, 2100 Copenhagen Ø, Denmark; 4 Department of Prevention and Documentation, Danish Cancer Society, Strandboulevarden 49, 2100 Copenhagen Ø, Denmark

**Keywords:** colorectal cancer, screening, socioeconomic position, participation

## Abstract

**Background:**

Colorectal cancer (CRC) screening with faecal occult blood test (FOBT) has the potential to reduce the incidence and mortality of CRC. Screening uptake is known to be inferior in people with low socioeconomic position (SEP) when compared with those with high position; however, the results of most previous studies have limited value because they are based on recall or area-based measures of socioeconomic position, and might thus be subject to selective participation and misclassification. In this study we investigated differences in CRC screening participation using register-based individual information on education, employment, and income to encompass different but related aspects of socioeconomic stratification. Also, the impact of ethnicity and cohabiting status was analysed.

**Methods:**

A feasibility study on CRC screening was conducted in two Danish counties in 2005 and 2006. Screening consisted of a self-administered FOBT kit mailed to 177 114 inhabitants aged 50–74 years. Information on individual socioeconomic status was obtained from Statistics Denmark.

**Results:**

A total of 85 374 (48%) of the invited returned the FOBT kits. Participation was significantly higher in women than in men (OR=1.58 (1.55–1.61)), when all socioeconomic and demographic variables were included in the statistical model. Participation also increased with increasing level of education, with OR=1.38 (1.33–1.43) in those with a higher education compared with short education. Also, participation increased with increasing income levels, with OR=1.94 (1.87–2.01) in the highest *vs* lowest quintile. Individuals with a disability pension, the unemployed and self-employed people were significantly less likely to participate (OR=0.77 (0.74–0.80), OR=0.83 (0.80–0.87), and OR=0.85 (0.81–0.89), respectively). Non-western immigrants were less likely to participate (OR=0.62 (0.59–0.66)) in a model controlling for age, sex, and county; however, this difference might be attributed to low SEP in these ethnic groups ((OR=0.93 (0.87–0.99), when adjusting for SEP indicators).

**Conclusion:**

This study based on individual information on several socioeconomic dimensions in a large, unselected population allowed for identification of several specific subgroups within the population with low CRC screening participation. Improved understanding is needed on the effect of targeted information and other strategies in order to reduce socioeconomic inequalities in screening.

Colorectal cancer (CRC) is the second most common cancer, and also the second leading cause of death from cancer in Europe (Ferlay *et al*, 2007). Randomised controlled trials have shown that screening with faecal occult blood test (FOBT) and subsequent identification and removal of symptom-free polyps and early stages of cancer can reduce the incidence and mortality of CRC significantly (Mandel *et al*, 1993; Kewenter *et al*, 1994; Hardcastle *et al*, 1996; Kronborg *et al*, 1996). A recent Cochrane review indicated that screening with FOBT has the potential to reduce CRC mortality by 16% (Hewitson *et al*, 2008). Colorectal cancer screening programmes have been organised in, for example, the United Kingdom, Finland, and France (Weller *et al*, 2007; Malila *et al*, 2008; Pornet *et al*, 2010).

In 2005 and 2006, a feasibility study was conducted in two Danish counties to evaluate whether a nationwide CRC screening programme should be implemented. CRC screening using FOBT or colonoscopy had by then not been offered in Denmark, and colonoscopies had been performed on medical indications only. Free FOBT test kits were mailed to the target population, who had to administer it at home by applying a small specimen of faeces on the test kit and forward it to the analysis centre, where the investigation of occult blood in the faeces was conducted. The clinical findings of the study were satisfactory, with 2.04 detected cancers per 1000 screened people and 64% of detected cancers being in the early stages. The participation rate was 48% (The Danish Centre for Health Technology Assessment, 2008), which is comparable with uptake rates from France (42%) (Pornet *et al*, 2010) and United Kingdom (52% in the second round of screening) (Weller *et al*, 2007), but substantially below uptake rates in Finland (71%) (Malila *et al*, 2008).

It is well known that uptake rates of CRC screening are higher among people with high socioeconomic position (SEP). Unequal uptake has the potential to widen an existing socioeconomic inequality in stage at diagnose and survival, which makes this issue an important study subject (Frederiksen *et al*, 2008; Mitry *et al*, 2008; Frederiksen *et al*, 2009). However, with a few exceptions, most previous studies are US-based studies, involving some kind of payment or they are based on questionnaires and are thus biased because of selective recall and participation (Cokkinides *et al*, 2003; Seeff *et al*, 2004; Wee *et al*, 2005; Peterson *et al*, 2007; Weber *et al*, 2008; Parente *et al*, 2009). Other studies analyse a sample of the invited population (Pornet *et al*, 2010) or use area-based measures of SEP (Weller *et al*, 2007; von Wagner *et al*, 2009a) as proxies for individual-level measures, which is likely to underestimate the true individual effect, but associations could be biased in either direction by the ecological fallacy (Galobardes *et al*, 2006a).

In the present study we were able to achieve information for each individual on a range of socioeconomic indicators on the total study population using the central Danish registers. We report on the three common indicators of SEP, income, occupation, and education, which can be linked to Weber's three dimension of social class (Liberatos *et al*, 1988), and encompass different, but related aspects of SEP. We aimed to investigate the influence of these SEP indicators on participation in an organised, systematic CRC screening programme using a self-administered approach, and more specifically to identify subgroups of the population less likely to participate.

## Materials and methods

### Study population and end point

**Figure 1:**
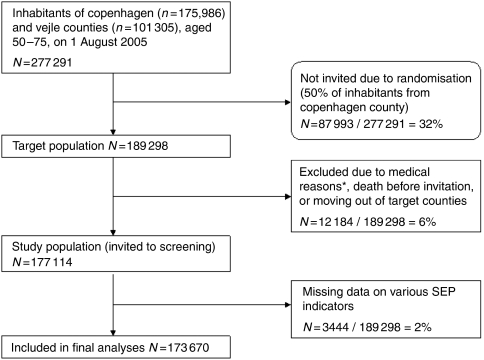
Flow diagram. ^*^History of previous colorectal cancer, adenomas, inflammatory bowel disease, or colonoscopy within the previous 2 years (in Vejle county only).

The study was conducted in the period August 2005 to December 2006. Participants were recruited from the two counties of Copenhagen and Vejle, with a total of 277 291 residents aged 50–74, on 1 August 2005 (Figure 1). The county of Copenhagen constitutes mainly urban areas surrounding the capital of Denmark, but includes some rural area. The county of Vejle is more rural, with three major provincial cities and many smaller or larger towns. In Copenhagen County, all residents aged 50–74 years were considered candidates, but only a random sample of half the population was invited. Additionally, cohabiting partners aged 50–74 years of those sampled were invited. In Vejle County, all residents aged 50–74 were invited to participate. In both counties, residents with a history of previous CRC, adenomas, or inflammatory bowel disease had been identified in a nation-wide central registry of pathology specimens and were excluded from the target population prior to invitation. Also, in Vejle County it was possible to identify people with a history of colonoscopy within the previous 2 years and exclude them. A total of 177 114 individuals (85 592 from Copenhagen and 91 522 from Vejle) were mailed a kit for FOBT, with a personal letter, and a prepaid reply envelope. Invited people were instructed on how to use the test through an enclosed leaflet with instructions and an educational section. In communities with a high percentage of inhabitants of non-Danish origin, the letter also included multi-lingual instruction, and an opportunity to get information by telephone was offered. Two reminder letters were sent to nonresponders. The first reminder letter included a test kit and was mailed after 2 months. The second reminder letter, which was mailed after another 6 weeks, included only the invitation and information on how to get a new test kit. Responders returned the test kits to central units, whereby exact registration of participation was obtained.

### Socioeconomic and demographic variables

The socioeconomic data on the entire study population were derived by linkage to the Central Population Registry and the population-based Integrated Database for Labour Market Research (IDA) in Statistics Denmark, using a unique personal ten-digit identifier, which is given to all people staying in Denmark for >3 months. Thus, information on age, sex, ethnicity, place of residence, education, cohabitation status, employment, and income were obtained for each invited person. For this study, ethnicity was categorised as Danish, immigrants, or descendants from western countries (the member states of the European Union (as of 31 December 2003), Andorra, Australia, Canada, Iceland, Liechtenstein, Monaco, New Zealand, Norway, San Marino, Switzerland, Vatican State, and the United States), and immigrants or descendants from non-western countries (all other countries). Education was categorised into three groups as short education (i.e., mandatory education of up to 7 years), medium education (up to 12 years – latest grades of primary school, secondary school, and vocational education), and higher education (>12 years). Cohabitation status was categorised as ‘single’ or ‘living with partner’. Employment specifies the characteristic of the most important employment during the year of screening and grades employed people according to the level of skills required to carry out their job. Income was defined as household income, adjusted for the number of people in the household and deflated according to the 2000 value of the Danish crown. Yearly variation in income was accounted for by calculating the average income in the 5 years before the diagnosis.

### Statistical methods

Differences in the distribution of variables by level of participation were analysed using the *χ*^2^ test. Multivariate logistic regression models were used to examine the influence of the socioeconomic factors on participation using the LOGISTIC procedure of SAS 9.1.3 (SAS Institute Inc., Cary, NC, USA). A two-step model was used. In the baseline model, each socioeconomic variable was entered alone and adjusted for age, sex, and county. In the mutually adjusted model, all variables were included. For each model, the odds ratio (OR) and 95% confidence intervals (CIs) were calculated. All tests were two sided. Investigation of interaction between sex and the other covariates, and between county and the other covariates, was performed. Statistical interactions were found in all cases, and therefore analyses stratified by sex and county were performed. These analyses were, however, very similar to the results of the analyses on the total sample, and therefore only main effects are shown in this paper. The stratified analyses are presented in Supplementary Table 1 on the webpage (http://www.nature.com/bjc). Analyses were checked for collinearity, which was not found. Model fits were tested by the Hosmer–Lemeshow *χ*^2^ test with the data grouped in percentiles of the fitted values.

## Results

Of the 177 114 individuals invited to CRC screening, 85 374 returned the test kit, giving a participation rate of 48%. Table 1 (column 1) gives rates of participation, and shows that inhabitants in the more rural county of Vejle were more likely than those from the county of Copenhagen to participate in the screening. Furthermore, responders were more likely to be female, to be of Danish or Western origin, to have had higher education, to be living with a partner, and to have a higher income than nonresponders.

The multivariate logistic regression analyses showed that the odds of participation increased with increasing age until the age of 70, after which a reduction was seen in uptake among those aged 70–74. Women were significantly more likely to participate than men (OR=1.45 (1.42–1.48), baseline model, column 2), as were inhabitants from Vejle when compared with inhabitants from Copenhagen (OR=1.25 (1.23–1.28)). The estimates did not change considerable in the final model, column 3. The odds of participation were nearly twice as low among non-western individuals as for Danes (OR=0.62 (0.59–0.66)). However, the association between ethnicity and participation was severely attenuated after adjustment for the socioeconomic factors in the final model (OR=0.93 (0.87–0.99)). This suggests that the ethnic difference may be attributed to differences in SEP among the ethnic groups. There was increasing odds of participation with increasing level of education, with an OR of 1.88 (1.83–1.94) among invited with a higher education compared with those with only obligatory education. The educational estimates were somewhat attenuated by including the other SEP variables in the final model, but still significant (OR=1.38 (1.33–1.43)). Also, individuals living alone were less likely to participate when compared with those living with a partner (OR=0.64 (0.63–0.65)). Controlling for other SEP variables in the final model attenuated this estimate slightly. Also, employment had a significant impact. The odds of participation were two times lower for those with a disability pension as for wage earners with a job requiring basic skills. When adjusting for level of education, cohabitation status, and income in the final model, the association attenuated substantially, but was still significant (OR=0.77 (0.74–0.80)). Old-age pensioners were also less likely to participate (OR=0.81 (0.77–0.85)); however, in the final model their odds were increased compared with the reference group (OR=1.13 (1.07–1.19)). This change in the estimate was primarily caused by including income in the model. Furthermore, the baseline as well as the final model showed that the unemployed and self-employed were less likely to participate, whereas pensioners on voluntarily early retirement had a higher rate of participation than did basic wage earners (OR=1.37 (1.31–1.42)). A gradient in participation was seen with increasing income; individuals within the highest income quintile being two times more likely to participate in FOBT screening than those with the lowest income quintile.

## Discussion

This register-based study analysed the effect of several individual socioeconomic factors on participation in systematic CRC screening on a large population of 177 114 individuals. We showed that low SEP, as measured by education, employment, and income, was strongly associated with low participation in testing for faecal occult blood, and that non-western immigrants were less likely to participate; however, this gradient was probably attributed to lower SEP in the ethnic groups.

Our results are in accordance with previous European studies; however, these have some methodological dissimilarities when compared with the present study. A study on inequalities in participation in the first round of the national CRC screening programme in England used an area-level deprivation score based on postcode sectors, and found a linear association between quintiles of deprivation and the return of test kits; uptake in the most affluent quintile being 50% higher than in the most deprived quintile (von Wagner *et al*, 2009a). In this study, test kits were mailed to the target population as in ours, whereas in a study from the French geographical department of Calvados, the target population was invited by post to consult the general practitioner (GP) of their choice to obtain test kits that were to be conducted at home (Pornet *et al*, 2010). Using a multilevel approach on a representative sample of the invited population, the French study found that after adjustment for individual factors (age, sex, insurance coverage, and Townsend score), participation was lowest in the most deprived neighbourhoods. No significant influence of GP density was observed. In an Italian study, test kits were distributed by pharmacists. Responses to mailed questionnaires among a subsample of 400 individuals from the target population indicated that high levels of education and non-manual work were positively associated with participation; however, the analyses were unadjusted (Parente *et al*, 2009). Higher uptake rates in the most affluent individuals have also been documented in several US-based studies based on self-report of previous screening activity (Cokkinides *et al*, 2003; Seeff *et al*, 2004; Wee *et al*, 2005; Peterson *et al*, 2007; Weber *et al*, 2008).

In the present study, FOBT kits were mailed out, and the barrier of visiting the health staff was thus bypassed. Mailed test kits may be an advantage, as it makes it possible to target all individuals and it may be more practical and less time consuming for the participants. On the other hand, information about screening and its implications is given in written form only, which may be a disadvantage for less educated individuals. A recent UK study showed that reliance on printed communication when inviting low-literate adults for CRC screening can be problematic (von Wagner *et al*, 2009b). Also, previous studies have demonstrated that the knowledge of CRC in the general population is low, and that knowledge is lowest in the less educated (Pullyblank *et al*, 2002; Keighley *et al*, 2004; Sessa *et al*, 2008). Distributing FOBT test kits through the GP may have the advantage that the GP will be able to discuss pros and cons with the patient prior to testing.

In the present study the wealth of detail in the central socioeconomic registers allowed us to analyse whether particular subgroups were less likely to participate in the screening study. We found that the odds of participation were halved among those with the shortest education, low income, and those on disability pension compared with the respective reference groups, and were also reduced among the unemployed, self-employed, and old-age pensioners. When adjusting for income, old-age pensioners were, however, more likely to participate than the basic wage earners. The inferior participation in these groups may be interpreted within the frame of the Health Belief Model, relating participation to perceived susceptibility, severity, threats, benefits, barriers, cues to action, and self-efficacy (Janz *et al*, 2002). Low levels of knowledge may lead to lower perceived susceptibility. Low SEP individuals, and in particular those on disability pension, may perceive less benefit of screening, because of competing daily social or health difficulties, whereas the self-employed may be more harassed. The inferior participation in the youngest and oldest age groups, as also seen in other studies (UK Colorectal Cancer Screening Pilot Group, 2004; Denis *et al*, 2007; Pornet *et al*, 2010), may be caused by a minor level of perceived threat of cancer in the former, and a perception that the benefits are small in the elderly, maybe struggling with general debility and comorbidities. Other barriers to CRC screening include worry about germs or contamination in completing the FOBT, which is more dominant among the less educated (James *et al*, 2008), as well as fear of test results and general aversion to screening (Parente *et al*, 2009). Also, the low CRC screening uptake in low SEP individuals must be interpreted in line with several other health-promoting activities with low compliance among these groups. Studies have shown that low use of CRC screening is associated with health behaviours such as smoking, low intake of fruit and vegetables, low levels of physical activity, and infrequent seat belt use (Shapiro *et al*, 2001; Seeff *et al*, 2004; Weber *et al*, 2008), which is more common among low SEP people. Also, screening for cervical and breast cancer is less frequent in these groups (von Euler-Chelpin *et al*, 2008; Moser *et al*, 2009). Health-promoting activities in general are more rapidly taken up by high SEP people. These aspects have implications for cancer survival among social groups.

Improvements in cancer survival over the last decades have been widely observed (Coleman *et al*, 2004; Storm *et al*, 2008; Edwards *et al*, 2010). The United States has experienced a fall in the CRC incidence rate of 22% and a decline by 26% in the CRC death rate from 1975 through 2000. Half of the reductions have been attributed to expanded use of effective screening tests (Edwards *et al*, 2010). Also, implementation of national CRC screening programmes in the European countries are likely to result in such improvements because of earlier detection of tumours, leading to better treatment options and higher survival. However, an increase in the relative socioeconomic inequality in the incidence and survival of CRC seems inevitable because of unequal participation. In the United States, successful initiatives to increase screening colonoscopy among urban minorities have used ‘patient navigators’ (Chen *et al*, 2008), whereas in England regional cancer intelligence units have special programmes to increase screening uptake in deprived groups (http://www.wmpho.org.uk/wmciu/).

Our study has both strengths and limitations. First, the study is based on a large sample covering 18% of the Danish population aged 50–74 years, who had complete information on follow-up. This is an advantage compared with data in studies based on recall of previous screening history, which may be prone to both selection and recall bias (Cokkinides *et al*, 2003; Seeff *et al*, 2004; Wee *et al*, 2005; Peterson *et al*, 2007; Phillips *et al*, 2007; Weber *et al*, 2008). Second, our analyses were done using individual data on SEP, thereby reducing misclassification of exposure, which arises when using area-based measures of SEP (Galobardes *et al*, 2006b). Furthermore, SEP data were achieved from central registers, which collect data prospectively and for administrative purpose, thus eliminating recall bias as often seen when using SEP data based on self-report (Turrell, 2000). A minor weakness of the study is that information on education was missing in 2% of those invited, whereas that figure was 20% for the subgroup of non-western immigrants. However, it is not likely that the missing data have influenced the major conclusions.

Colorectal cancer screening has been documented to reduce mortality and is now being implemented in a large number of countries. Analyses from the present study population have shown that cost effectiveness is not affected by low participation rates if >40% (The Danish Centre for Health Technology Assessment, 2008). However, our results demonstrate that although much effort has been made to inform those to be screened, CRC screening does not cover the entire population, and especially not those with low SEP. More efficient methods need to be developed to make CRC screening socially well balanced.

## Figures and Tables

**Table 1:** Descriptive data and ORs measuring the association between socio-demographic factors and participation in systematic CRC screening with FOBT among 50–74-year-old inhabitants of two Danish counties (*N*=177 114)

		**Baseline model**	**Final model**
	**No. (%) of responders, 85 374 (48.2)**	**OR (95% CI) adjusted for age, sex, and county**	**OR (95% CI) mutually adjusted** a
*Age*			
50–54	17 383 (45.2)^b^	1	1
55–59	21 557 (48.6)^b^	1.15 (1.12–1.18)	1.21 (1.18–1.24)
60–64	20 486 (50.8)^b^	1.26 (1.22–1.29)	1.37 (1.33–1.42)
65–69	15 314 (51.4)^b^	1.28 (1.24–1.32)	1.53 (1.47–1.60)
70–74	10 634 (44)^b^	0.94 (0.91–0.97)	1.28 (1.21–1.36)
			
*Sex*			
Male	37 556 (43.6)^b^	1	1
Female	47 818 (52.6)^b^	1.45 (1.42–1.48)	1.58 (1.55–1.61)
			
*County*			
Copenhagen	38 886 (45.4)^b^	1	
Vejle	46 488 (50.8)^b^	1.25 (1.23–1.28)	1.29 (1.26–1.31)
			
*Ethnicity*			
Danish	81 187 (48.7)^b^	1	
Western	1975(46.9)^b^	0.96 (0.90–1.02)	0.96 (0.90–1.02)
Non-western	2152 (35.7)^b^	0.62 (0.59–0.66)	0.93 (0.87–0.99)
Missing	60 (43.2)^b^		
			
*Education*			
Short	16 354 (42.6)	1	
Medium	45 192 (48.1)	1.39 (1.36–1.43)	1.19 (1.16–1.22)
Higher	22 596 (54.6)	1.88 (1.83–1.94)	1.38 (1.33–1.43)
Missing			
			
*Cohabitation*			
Living with partner	65 877 (51.0)^b^	1	1
Living alone	19 452 (40.1)^b^	0.64 (0.63–0.65)	0.72 (0.71–0.74)
Missing	45 (42.9)^b^		
			
*Employment*			
Self-employed	4254 (44.6)^b^	0.87 (0.83–0.91)	0.85 (0.81–0.89)
Wage earners, high level	18 581 (54.7)^b^	1.32 (1.28–1.35)	1.08 (1.04–1.11)
Wage earners, basic level	24 224 (48.9)^b^	1	1
Old-age pension	16 835 (45.9)^b^	0.81 (0.77–0.85)	1.13 (1.07–1.19)
Voluntary early retirement pension	11 204 (56.5)^b^	1.10 (1.06–1.15)	1.37 (1.31–1.42)
Disability pension	5296 (34.6)^b^	0.50 (0.48–0.52)	0.77 (0.74–0.80)
Unemployed	4952 (40.4)^b^	0.68 (0.65–0.70)	0.83 (0.80–0.87)
Missing	28 (43.8)^b^		
			
*Income*			
1–24% percentile	16 810 (38.0)^b^	1	1
25–49% percentile	21 130 (47.7)^b^	1.61 (1.57–1.66)	1.44 (1.39–1.48)
50–74% percentile	23 265 (52.6)^b^	2.21 (2.14–2.28)	1.82 (1.76–1.88)
75–100% percentile	24 135 (54.5)^b^	2.50 (2.42–2.57)	1.94 (1.87–2.01)
Missing	34 (37.8)^b^		

Abbreviations: CI=confidence interval; CRC=colorectal cancer screening; FOBT=faecal occult blood test; OR=odds ratio.

a OR from multivariate logistic regression analysis including all variables in the table as covariates.

b The *χ*^2^ test <0.0001.

Model with main effects only.
