# Supplementary material for: Socioeconomic position and participation in colorectal cancer screening
Source: Br J Cancer. 2010 Oct 19;103(10):1496–501. doi: 10.1038/sj.bjc.6605962 (PMC2990593; doi:10.1038/sj.bjc.6605962)
Supplement: Supplementary Online Table [file 6605962x1.doc]

Supplementary online table

| Odds ratios measuring the association between socioeconomic factors and participation in systematic colorectal cancer screening with faecal occult blood test among 50-74 year old inhabitants of two Danish counties (N=177,114). Stratified by county and sex. Analyses are adjusted for age, ethnicity, education, cohabition, employment, and income. | | | | | | | | | | | | | | | |
| --- | --- | --- | --- | --- | --- | --- | --- | --- | --- | --- | --- | --- | --- | --- | --- |
|  | **All** | | | **Copenhagen** | | | | | | **Vejle** | | | | | |
|  |  |  |  | **Female** | | | **Male** | | | **Female** | | | **Male** | | |
|  | **N=177,114** | | | **N=44,958** | | | **N=40,634** | | | **N=45,973** | | | **N=45,549** | | |
|  | **OR** | LCL | UCL | **OR** | LCL | UCL | **OR** | LCL | UCL | **OR** | LCL | UCL | **OR** | LCL | UCL |
| **County** |  |  |  |  |  |  |  |  |  |  |  |  |  |  |  |
| Copenhagen | **1** |  |  | **1** |  |  | **1** |  |  | **0** |  |  | **0** |  |  |
| Vejle | 1.29 | 1.26 | 1.31 | **0** |  |  | **0** |  |  | **1** |  |  | **1** |  |  |
|  |  |  |  |  |  |  |  |  |  |  |  |  |  |  |  |
| **Sex** |  |  |  |  |  |  |  |  |  |  |  |  |  |  |  |
| Male | **1** |  |  | **0** |  |  | **1** |  |  | **0** |  |  | **1** |  |  |
| Female | 1.58 | 1.55 | 1.61 | **1** |  |  | **0** |  |  | **1** |  |  | **0** |  |  |
|  |  |  |  |  |  |  |  |  |  |  |  |  |  |  |  |
| **Age** |  |  |  |  |  |  |  |  |  |  |  |  |  |  |  |
| 50-54 | **1** |  |  | **1** |  |  | **1** |  |  | **1** |  |  | **1** |  |  |
| 55-59 | 1.21 | 1.18 | 1.24 | 1.20 | 1.13 | 1.27 | 1.18 | 1.11 | 1.25 | 1.27 | 1.20 | 1.34 | 1.18 | 1.12 | 1.25 |
| 60-64 | 1.37 | 1.33 | 1.42 | 1.24 | 1.16 | 1.32 | 1.34 | 1.25 | 1.43 | 1.40 | 1.31 | 1.49 | 1.47 | 1.38 | 1.56 |
| 65-69 | 1.53 | 1.47 | 1.60 | 1.24 | 1.14 | 1.35 | 1.55 | 1.42 | 1.69 | 1.47 | 1.34 | 1.62 | 1.79 | 1.65 | 1.95 |
| 70-74 | 1.28 | 1.21 | 1.36 | 0.91 | 0.81 | 1.03 | 1.53 | 1.37 | 1.72 | 1.09 | 0.96 | 1.23 | 1.67 | 1.49 | 1.86 |
|  |  |  |  |  |  |  |  |  |  |  |  |  |  |  |  |
| **Ethnicity** |  |  |  |  |  |  |  |  |  |  |  |  |  |  |  |
| Danish | **1** |  |  | **1** |  |  | **1** |  |  | **1** |  |  | **1** |  |  |
| Western | 0.96 | 0.90 | 1.02 | 1.04 | 0.93 | 1.15 | 1.02 | 0.90 | 1.16 | 0.86 | 0.73 | 1.00 | 0.87 | 0.75 | 1.01 |
| Non-Western | 0.93 | 0.87 | 0.99 | 0.82 | 0.74 | 0.91 | 0.97 | 0.87 | 1.07 | 0.84 | 0.71 | 0.99 | 1.27 | 1.08 | 1.49 |
|  |  |  |  |  |  |  |  |  |  |  |  |  |  |  |  |
| **Education** |  |  |  |  |  |  |  |  |  |  |  |  |  |  |  |
| Short | **1** |  |  | **1** |  |  | **1** |  |  | **1** |  |  | **1** |  |  |
| Medium | 1.19 | 1.16 | 1.22 | 1.24 | 1.17 | 1.30 | 1.26 | 1.18 | 1.34 | 1.11 | 1.06 | 1.17 | 1.19 | 1.13 | 1.25 |
| Higher | 1.38 | 1.33 | 1.43 | 1.45 | 1.35 | 1.55 | 1.49 | 1.38 | 1.61 | 1.26 | 1.18 | 1.35 | 1.31 | 1.23 | 1.40 |
|  |  |  |  |  |  |  |  |  |  |  |  |  |  |  |  |
| **Cohabition** |  |  |  |  |  |  |  |  |  |  |  |  |  |  |  |
| Living with partner | **1** |  |  | **1** |  |  | **1** |  |  | **1** |  |  | **1** |  |  |
| Living alone | 0.72 | 0.71 | 0.74 | 0.90 | 0.87 | 0.94 | 0.70 | 0.66 | 0.74 | 0.75 | 0.72 | 0.78 | 0.56 | 0.53 | 0.59 |
|  |  |  |  |  |  |  |  |  |  |  |  |  |  |  |  |
| **Employment** |  |  |  |  |  |  |  |  |  |  |  |  |  |  |  |
| Self-employed | 0.85 | 0.81 | 0.89 | 0.82 | 0.73 | 0.92 | 0.86 | 0.79 | 0.94 | 0.78 | 0.70 | 0.88 | 0.90 | 0.84 | 0.97 |
| Wage earners, high level | 1.08 | 1.04 | 1.11 | 1.09 | 1.02 | 1.16 | 1.13 | 1.06 | 1.21 | 0.96 | 0.89 | 1.03 | 1.10 | 1.04 | 1.18 |
| Wage earners, basic level | **1** |  |  | **1** |  |  | **1** |  |  | **1** |  |  | **1** |  |  |
| Old age-pension | 1.13 | 1.07 | 1.19 | 1.24 | 1.12 | 1.38 | 1.19 | 1.07 | 1.33 | 0.96 | 0.86 | 1.07 | 1.27 | 1.14 | 1.40 |
| Voluntary early retirement pension | 1.37 | 1.31 | 1.42 | 1.51 | 1.39 | 1.64 | 1.47 | 1.34 | 1.61 | 1.24 | 1.14 | 1.34 | 1.35 | 1.24 | 1.47 |
| Disablilty pension | 0.77 | 0.74 | 0.80 | 0.79 | 0.73 | 0.86 | 0.78 | 0.70 | 0.87 | 0.69 | 0.64 | 0.75 | 0.81 | 0.74 | 0.89 |
| Unemployed | 0.83 | 0.80 | 0.87 | 0.88 | 0.81 | 0.95 | 0.95 | 0.86 | 1.04 | 0.73 | 0.67 | 0.79 | 0.83 | 0.75 | 0.92 |
|  |  |  |  |  |  |  |  |  |  |  |  |  |  |  |  |
| **Income** |  |  |  |  |  |  |  |  |  |  |  |  |  |  |  |
| 0-24% percentile | **1** |  |  | **1** |  |  | **1** |  |  | **1** |  |  | **1** |  |  |
| 25-49% percentile | 1.44 | 1.39 | 1.48 | 1.45 | 1.37 | 1.53 | 1.48 | 1.39 | 1.59 | 1.34 | 1.27 | 1.42 | 1.52 | 1.43 | 1.62 |
| 50-74% percentile | 1.82 | 1.76 | 1.89 | 1.74 | 1.63 | 1.86 | 1.92 | 1.78 | 2.07 | 1.69 | 1.58 | 1.80 | 2.08 | 1.93 | 2.23 |
| 75-100% percentile | 1.94 | 1.87 | 2.01 | 1.85 | 1.72 | 1.99 | 2.06 | 1.89 | 2.23 | 1.83 | 1.70 | 1.97 | 2.21 | 2.05 | 2.39 |
| OR = Odds Ratio, LCL = Lower confidence limit, HCL = Higher confidence limit | | | | | | | | | | | | | | | |
